# Real-time *in vivo* imaging of regional lung function in a mouse model of cystic fibrosis on a laboratory X-ray source

**DOI:** 10.1038/s41598-019-57376-w

**Published:** 2020-01-16

**Authors:** Rhiannon P. Murrie, Freda Werdiger, Martin Donnelley, Yu-wei Lin, Richard P. Carnibella, Chaminda R. Samarage, Isaac Pinar, Melissa Preissner, Jiping Wang, Jian Li, Kaye S. Morgan, David W. Parsons, Stephen Dubsky, Andreas Fouras

**Affiliations:** 1 Department of Mechanical and Aerospace Engineering, Monash University, Melbourne, Australia; 2 Robinson Research Institute and Adelaide Medical School, University of Adelaide, Adelaide, Australia; 3 Women’s and Children’s Hospital, North Adelaide, Australia; 4 Infection & Immunity Program, Monash Biomedicine Discovery Institute, and Department of Microbiology, Monash University, Melbourne, Australia; 5 4Dx Limited, Melbourne, Australia; 6 School of Physics and Astronomy, Monash University, Melbourne, Australia

**Keywords:** 3-D reconstruction, X-ray tomography, Respiration, Biomedical engineering, X-rays

## Abstract

Most measures of lung health independently characterise either global lung function or regional lung structure. The ability to measure airflow and lung function regionally would provide a more specific and physiologically focused means by which to assess and track lung disease in both pre-clinical and clinical settings. One approach for achieving regional lung function measurement is via phase contrast X-ray imaging (PCXI), which has been shown to provide highly sensitive, high-resolution images of the lungs and airways in small animals. The detailed images provided by PCXI allow the application of four-dimensional X-ray velocimetry (4DxV) to track lung tissue motion and provide quantitative information on regional lung function. However, until recently synchrotron facilities were required to produce the highly coherent, high-flux X-rays that are required to achieve lung PCXI at a high enough frame rate to capture lung motion. This paper presents the first translation of 4DxV technology from a synchrotron facility into a laboratory setting by using a liquid-metal jet microfocus X-ray source. This source can provide the coherence required for PCXI and enough X-ray flux to image the dynamics of lung tissue motion during the respiratory cycle, which enables production of images compatible with 4DxV analysis. We demonstrate the measurements that can be captured *in vivo* in live mice using this technique, including regional airflow and tissue expansion. These measurements can inform physiological and biomedical research studies in small animals and assist in the development of new respiratory treatments.

## Introduction

The early diagnosis and ongoing monitoring of chronic lung diseases, such as chronic obstructive pulmonary disease (COPD), pulmonary fibrosis, cystic fibrosis (CF), asthma and lung cancer, is currently hampered by the inability to capture the complete spatial distribution of lung function^1^. Changes in pulmonary function are traditionally quantified clinically through global lung health measures such as pulmonary function tests, which produce whole-lung parameters such as FEV1 or Lung Clearance Index (LCI). Since they are measured at the mouth these tests are unable to accurately localise where in the lung any change in function originates. They can also be age- and effort-dependent, and their sensitivity means they can often only detect abnormalities in the advanced stages of disease.

Imaging modalities like high-resolution computed tomography (CT) can be used to detect structural changes such as bronchiectasis, bronchial wall thickening, mucus plugging and air trapping, but in clinical practice CT is typically combined with other non-invasive pulmonary function testing such as spirometry, forced oscillation technique (FOT), plethysmography, or multiple breath wash in/out techniques. CT has also been combined with other techniques used to investigate thoracic dynamics^2^, such as the relation of diaphragmatic electric activity on lung aeration and collapse^3^, however this does not show localised lung dynamics or airflow. Disease location can be inferred via local assessment of structure such as with the PRAGMA-CF protocol^4^, but this technique extrapolates regional lung dysfunction through quantification of structural information captured from CT. Studies have also attempted to measure dynamic lung motion in humans by comparing CT scans at different respiratory phases (e.g. end-inspiratory and end-expiratory), to measure tissue deformation for tumour identification and tracking^5–7^. However, this type of technology has not become common clinical practice, and has not been widely utilised outside of tumour motion evaluation. Importantly, quantification of dynamic airflow with high resolution 3-dimensional spatial mapping is not yet available in clinical practice.

High-resolution CT imaging is now commonplace in small animals, enabling the identification of *structural* changes. However, pre-clinical studies of obstructive lung diseases (e.g. cystic fibrosis and asthma) in animal models also often lack robust *in vivo*, non-invasive techniques capable of identifying the location of *functional* deficits at high resolution (i.e. < 200 µm), and how they impact lung function. Typically, read-outs of lung function in these studies are global – such as airway resistance and compliance from lung mechanics testing using devices such as the flexiVent small animal ventilator (Scireq, Canada) – or they require euthanasia and post-mortem analysis of lung tissue using histological or biochemical tests to detect and localise disease. For longitudinal studies, the requirement for euthanasia means additional animals must be used for each time point, adding to the cost of such studies, and increasing the variability in results between animals. An ideal assessment technique would be able to obtain dynamic functional measures such as airflow in a regional manner to identify the location of any functional changes, and understand how they contribute to global changes in parameters such as FEV1.

To attempt to capture dynamic motion in pig lungs, Perchiazzi (2014) performed CT scans during multiple inspiratory hold manoeuvres at increasing pressures to quantify regional compliance^8^. However, because the motion of the lung parenchyma could not be tracked they used a registration algorithm to quantify the motion of manually identified landmarks at the edge and within transverse slices. Using this method they could create regional lung compliance maps. The methodologies we describe in this manuscript use custom designed hardware and software to enable two key evolutions: (1) Use of phase-contrast X-ray imaging to visualise the motion of the lung parenchyma in live mice on a laboratory source, and (2) performing CT acquisition during ventilated breathing without breath holds allows capture of the natural motion of the lung during respiration. Thus, we aim to use X-ray imaging methods to achieve effective, high-resolution *in vivo* data about the dynamic function of the lung. As in clinical studies, the availability of these methods in pre-clinical research would allow tracking of regional respiratory disease progression or response to therapies over time in the same animal, reduce animal numbers, improve statistical power, and provide additional information to researchers that is not currently available.

Phase-contrast X-ray imaging (PCXI) is capable of imaging soft tissues by enhancing the contrast of biological interfaces, a result of variations introduced to the phase of the X-ray wave by different materials. PCXI of the dense alveolar clusters in lung tissue produces a distinctive ‘speckle’ pattern^9^, and the motion of the speckle pattern during ventilation can be tracked via a technique known as X-ray velocimetry (XV)^10^. Lung expansion is a result of the volume of the thoracic cavity increasing by motion of the diaphragm, which causes air to flow through the bronchial airway tree inflating the lung tissue. As such, it is desirable to measure the regional lung tissue expansion and the airflow through each airway. Since XV can track lung tissue displacement and can be applied to 3-dimensional datasets (e.g. PCXI-CT), it can be used to capture lung expansion and contraction throughout the breath; a process we have termed 4DxV (with time being the fourth dimension). From these measurements the regional air volume in the lungs can be determined at multiple points throughout the breath. If this data is then associated with the airway tree structure, the time-varying airflow through each branch segment in the airway tree can be quantified^11^.

Structural changes from obstructive lung diseases such as asthma, COPD, emphysema and CF will alter the airflow within the bronchial tree and change the 4DxV lung expansion map, allowing poorly ventilated regions of the lung to be located. Restrictive lung diseases such as pulmonary fibrosis and interstitial lung disease, which can have a more widespread effect on the lung parenchyma and are often characterised by changes in lung tissue or chest wall stiffness, will also change the way the lungs expand on inhalation^12,13^. The 4DxV maps allow those regions of altered expansion to be identified^14^. The ability to monitor changes in local airflow is likely to be a useful indicator of disease progression or treatment effectiveness in many lung diseases.

The 4DxV functional lung imaging techniques were developed and validated using synchrotron radiation facilities^11^. For example, we have previously used 4DxV at the SPring-8 Synchrotron to demonstrate that β-ENaC mice – a recognised model of CF-like lung disease^15^ – exhibit patchy lung disease, with the expiratory time constant used as a measure of regional lung function^16^. However, for this technique to be widely-adopted as a research tool, and move towards clinical diagnostic imaging, a more compact and accessible set-up is required^17^. Liquid-metal-jet anodes are a recent advance in laboratory X-ray source technology that reduce the impact of heat-load restriction associated with solid metal anodes, therefore providing both small spot sizes and relatively high flux^18,19^. Previous studies have shown that this small spot size provides sufficient coherence to produce PCXI lung speckle^20^, and that PCXI-CT of the lungs in post-mortem animals is achievable for structural characterization^21–23^.

The aim of the present experiment was to demonstrate the ability to visualise dynamic regional ventilation and airflow *in vivo* on a custom-built laboratory 4DxV system, using normal and β-ENaC mice. Our results demonstrate the successful translation of these techniques to a laboratory PCXI system, with the high speed, dynamic and functional imaging capabilities required to image live mice during ventilation. The ability to study lung diseases in small animal models using 4DxV technology has great potential; modern gene editing techniques have facilitated the cost-effective creation of new respiratory models, enabling cost-effective respiratory therapeutics testing and physiology studies to be performed with high-throughput. Furthermore, a 2D version of the lung health assessment method developed and applied in these pre-clinical models is currently under review by the USA FDA under the 510(k) Premarket Notification scheme for use in humans to diagnose respiratory conditions in the clinic.

## Results and Discussion

**Figure 1:**
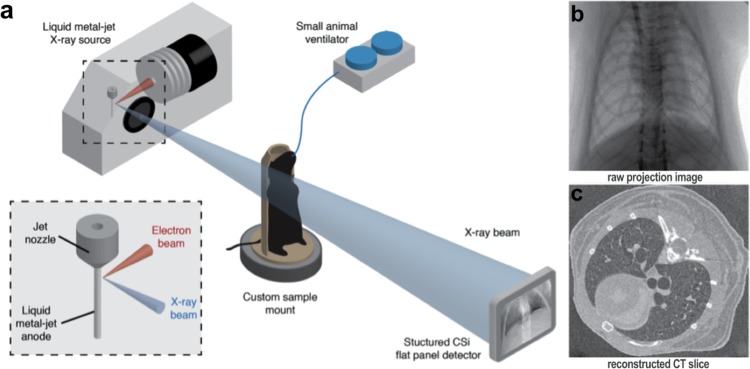
(**a**) Experimental image acquisition setup. Propagation-based phase contrast X-ray images of mouse lungs were acquired on a laboratory imaging setup consisting of a high brightness X-ray source with a liquid-metal-jet anode. Mice were non-surgically intubated and mechanically ventilated, then placed in a custom-built sample holder on a rotating stage for imaging. (**b**) Raw 2D projections were acquired over 360°, with images binned according to their time-point within the breath cycle. (**c**) CT volumes at each of the 15 time-points throughout the breath were then reconstructed from the binned projections, to produce a complete 4D CT dataset.

We present dynamic 4DxV *in vivo* images of the lungs and airways of live mice acquired on a laboratory X-ray source at 30 frames per second. All images were acquired at the Laboratory for Dynamic Imaging at Monash University on a propagation-based phase contrast X-ray imaging setup that uses an Excillum D2 + X-ray source (Excillum AB, Kista, Sweden)^24^. Mice were anaesthetised, non-surgically intubated, and ventilated, and then 4-dimensional PCXI-CT images were acquired and processed to provide airway and lung measurements. These images are a volumetric CT that captures projection images of the entire lung in the frame. Figure 1 shows the imaging setup, with experimental and post-processing details located in the Methods sections.

The radiation dose at a source power of 265 W and a 60 × 15 μm spot size was 7.2 mGy/s. The scans described here delivered between 1.47–1.74 Gy, well below the dose required to damage lung tissue and the lethal dose measure (LD_50/30_) which is approximately 7.5 Gy for BALB/C mice and 8.3 Gy for C57BL/6 mice^25^ (note previous work at a synchrotron delivered 10–15 Gy^11^). The resolution of the system was suitable for segmenting 60 µm diameter airways.

**Figure 2:**
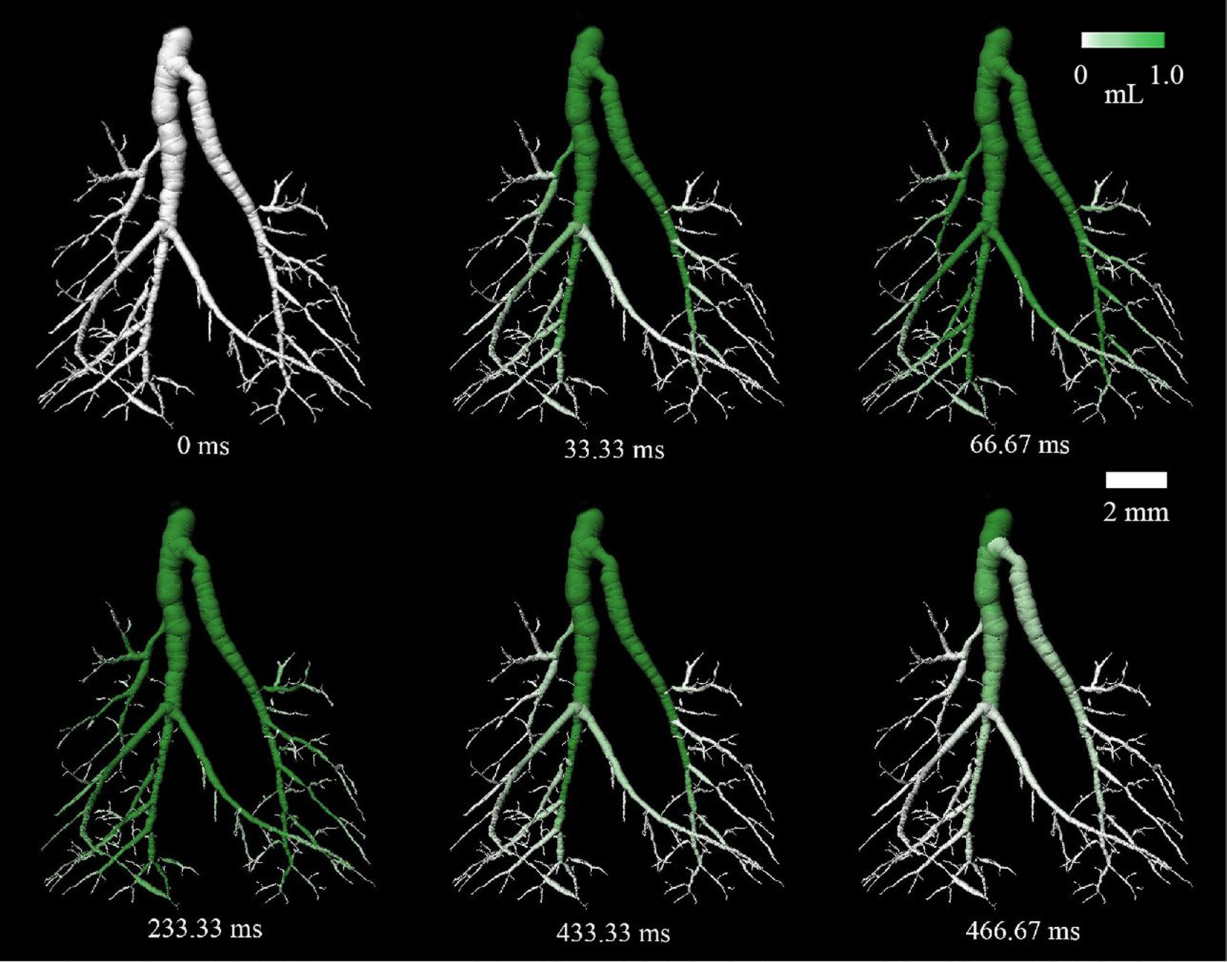
Aeration of the bronchial tree in a normal Swiss mouse. The white-green scale bar shows the measured air volume, relative to end-expiration, that has passed that location in the airway tree until that time point in the breath. This figure also demonstrates the high resolution of the airway tree branches achievable on a laboratory imaging system. Note that only six of the fifteen available time-points are shown here.

Figure 2 shows a visual representation of the air distribution within the airway tree of a normal Swiss mouse throughout the breath cycle. At the start of the breath (t = 0 ms) the volume of air in the lungs is the functional residual capacity, and then air begins to flow into the trachea and first bronchial bifurcation (t = 33 ms). At mid-inspiration (t = 66 ms) the primary airway branches are aerated and air begins to flow into the lower generation branches. At peak inspiration (t = 233 ms) the airways are maximally aerated and fresh air is supplied to the alveoli. At mid-expiration (t = 433 ms) the air is expelled from the lower generation branches during exhalation. Finally, close to the end of the breath (t = 466 ms) the airways return toward their relaxed state. The progression of balanced aeration at each bifurcation of the bronchi at each phase of the breath shows that there are no obstructions present in the airway tree of this normal animal. The movement of the air volume to the lowest generation of airways measured (the 6^th^ generation) shows uniform aeration to each region of each lobe of the lungs.

**Figure 3:**
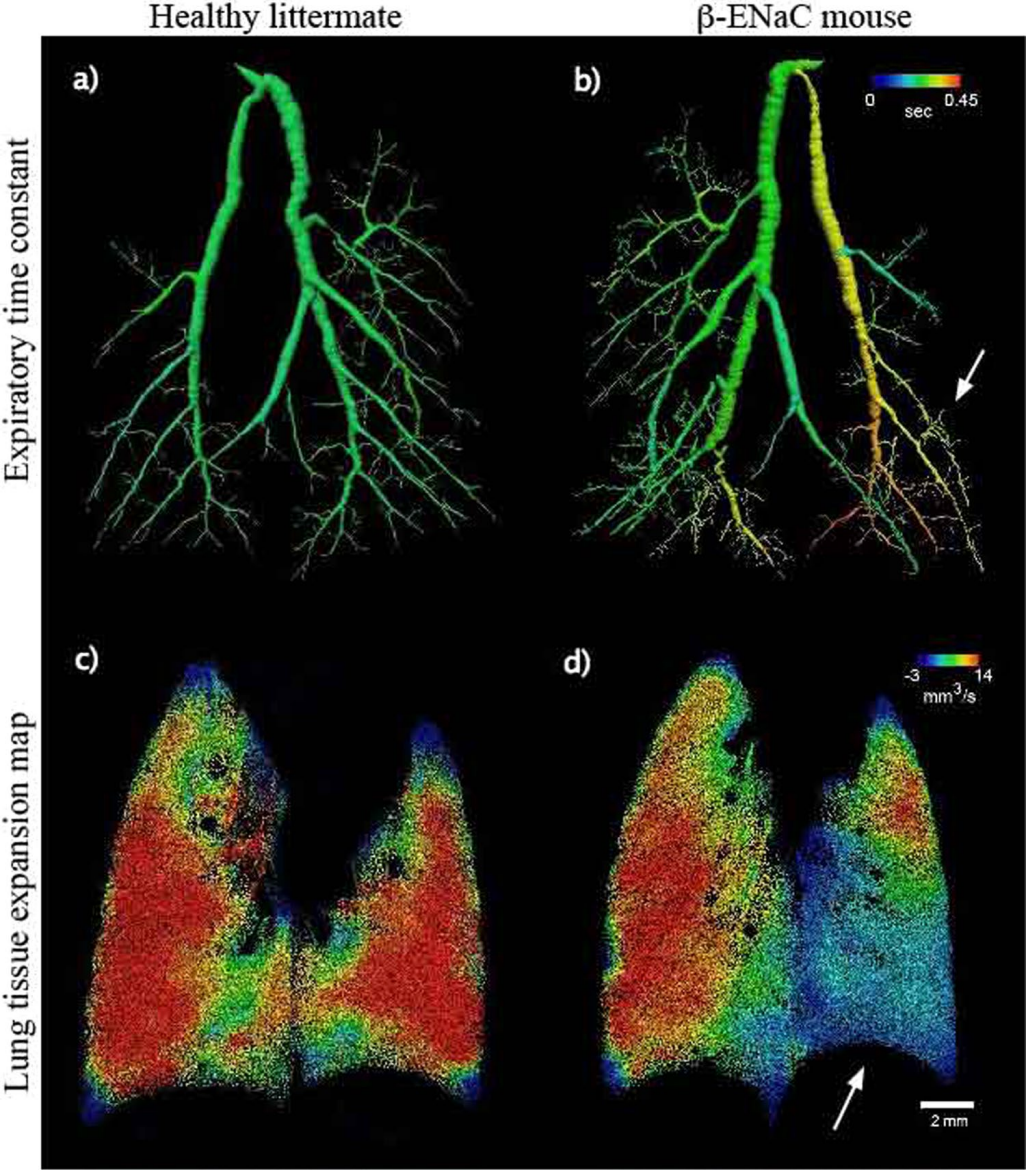
Two-dimensional projection images showing lung function analysis in a model of CF lung disease. (**a**,**b**) Airway segmentation from the 4DxV analysis of a healthy littermate and β-ENaC mouse, respectively, coloured by the local expiratory time constant. The lower part of the left anatomical lobe of the β-ENaC mouse shows an increased expiratory time constant (arrow), compared to both the right anatomical lobe and the littermate mouse. (**c**,**d**) The corresponding lung tissue expansion at the peak of the breath shows a clear reduction in expansion evident in the lower right anatomical lobe of the β-ENaC mouse (arrow), in comparison to the healthy littermate, in which the lung tissue expansion appears more uniform. The 4DxV expansion map around the heart is sparse due to cardiac motion artefacts.

Figure 3 shows the results of the 4DxV analysis on the lungs of a healthy littermate control (left) and a diseased β-ENaC mouse (right). The expiratory time constant of the airflow through the airway tree (top row), and the corresponding 4DxV lung tissue maximal expansion maps (bottom row) demonstrate that the flow of air from the supplying branches directly impacts the aeration of the lung tissue. The healthy control mouse clearly exhibits uniform aeration throughout the airway tree (Fig. 3a), which translates to homogeneous expansion (and thus aeration) of the lung tissue in both left and right lung lobes (Fig. 3c). In a healthy lung (e.g. Fig. 3c) the centre of each lobe expands the most, as this region contains the alveoli that are closest to the airway branches that supply the fresh air. Alveoli on the edges of the lobes are supplied by the smallest terminal airways furthest away from the primary branches, and thus aerate last, even in a healthy lung.

For the diseased β-ENaC mouse the expiratory time constant (Fig. 3b) shows that it takes longer for the air to be expelled from the left lung, with a higher local expiratory time constant (yellow/orange/red) seen for these airways likely due to the patchy CF-like obstructive airway disease present in this animal. In Fig. 3d, a dramatic reduction in regional expansion of the left lung is seen, as represented by the large light blue area. This directly correlates to reduced aeration for these regions of the lung, and thus a reduction in local lung function for this mouse.

## Conclusions

We have demonstrated the ability to perform *in vivo* 4DxV regional imaging of the lungs and airways in live mice, at 30 frames per second and at high resolution (60 µm) on a laboratory X-ray source, to obtain functional information on dynamic airflow. The dynamic process of airflow through the airway tree and the resulting expansion and ventilation of the lung tissue was measured using lung tissue motion measures from X-ray velocimetry. The data we acquired matches the findings from our previous 4DxV imaging studies using β-ENaC mice at SPring-8 Synchrotron^16^, where we found that β-ENaC animals exhibited patchy lung disease that was detectable via 4DxV, an increased regional time constant and mucus obstructed airways that matched the location of the functional defects (detected histologically by AB/PAS staining). This allows us to validate the successful translation of this technique to a custom-designed compact laboratory XV setup, which is a pivotal step in the pathway to preclinical and clinical application of this technique.

While the synchrotron set-up that we have used in other studies offers higher spatial resolution, stronger phase contrast and higher x-ray flux than this laboratory set-up, our custom-designed laboratory setup was still able to track the local motion and hence function of the lung. The laboratory source does deliver low-energy x-ray radiation that is not present at the synchrotron, which will contribute to the radiation dose without contributing significantly to image quality, however the magnification present at the laboratory set-up enables the use of a detector with larger pixels and a thicker scintillator, requiring less incident radiation (and hence dose) for the same number of detector counts.

The ability to obtain these high quality 4D CT and 4DxV lung images without the need for synchrotron access shows the likely impact this technology can have for *in vivo* studies on pulmonary disease physiology, disease detection and monitoring, and respiratory treatment effects. The ability to perform this technique in the laboratory makes longitudinal studies on disease progression and treatment response feasible, in a non-invasive and non-terminal way, at readily accessible and compact laboratory facilities - a task which is difficult at limited-access facilities, like a synchrotron. Future studies will examine larger cohorts of animals, and be designed to both understand disease and enable continued development of novel outcome assessment measures based on the 4DxV data.

Our study is a demonstration that a wealth of lung function information can be obtained from measures of lung motion in a laboratory setting. These 4DxV techniques are a novel tool that will enable localised quantification of a range of respiratory diseases to be made in small animal models, and in the future will likely facilitate advances in patient diagnosis, treatment and monitoring.

## Methods

### Experimental procedure

All experiments were approved by the Monash University Animal Ethics Committee and conformed to the guidelines set out in the NHMRC Australian Code of Practice for the Care and Use of Animals for Scientific Purposes. All animals were supplied by the Monash Animal Research Platform.

The mouse used to demonstrate airway segmentation and airflow (Fig. 2) was a 9 week-old female Swiss mouse. The mouse was anaesthetised with isoflurane and orotracheally (non-surgically) intubated. Medical grade oxygen containing isoflurane (2%) was continually administered through a small animal ventilator (Accuvent 200, Notting Hill Devices, Melbourne, Australia) to maintain anaesthesia.

The pair of mice shown in Fig. 3 included one 8 week old β-ENaC-Tg female and one 8 week old female littermate control (β-ENaC negative). β-ENaC over-expressing mice are a well-established model of cystic fibrosis lung disease as they overproduce mucus that obstructs airways^15^. Mice were anaesthetised by intraperitoneal (i.p.) injection with a mixture of 150 mg/kg ketamine (Parnell Australia Pty Ltd, Alexandria NSW, Australia) and 10 mg/kg xylazine (Xylazil-20, 60 Troy Laboratories Pty Ltd, Smithfield NSW, Australia), orotracheally intubated, and ventilated.

In all cases, mice were mounted vertically in a custom 3D-printed mouse holder for image acquisition, and the ventilation settings found in Table 1 were applied. Mice were humanely killed at the completion of imaging.

**Table 1:** Ventilation and imaging parameters used in the airway analysis imaging.

Figure	PIP (cm H_2_O)	PEEP (cm H_2_O)	Inspiratory/expiratory time (ms)	Respiratory rate (breaths/min)	Time points per breath	Projections per time point	Total number of projections over 360°	Radiation dose (mGy/s)
2	12	2	250/250	120	15	400	6000	~7
3	12	2	150/350	120	15	480	7200	~6

### Imaging procedure

All imaging was performed in the Laboratory for Dynamic Imaging at Monash University (Melbourne, Australia)^24^. The imaging setup can be seen in Fig. 1, and consisted of a high brightness X-ray source (Excillum AB, Kista, Sweden) with a liquid-metal-jet anode and a characteristic peak of 24 keV, as per the manufacturer data sheet^21^. Imaging was performed at 70 kVp with a 15 μm spot size and at a power of 265 W. A high-speed CMOS flat-panel detector (PaxScan, Varian Medical Systems, Palo Alto, CA, USA) with an isotropic pixel size of 194 μm was used to capture images at 30 frames per second (fps) with an 18 ms exposure. The source-to-isocenter of the rotational imaging stage (R_1_) and source-to-detector (R_2_) distances were 0.36 m and 3.0 m respectively, resulting in an effective pixel size of 20 μm. A 2.7 m vacuum tube in front of the detector reduced image noise due to scattering in air. The radiation dose was measured using a dosimeter (Fluke Biomedical, TNT12000 DoseMate, USA). A high-precision rotation stage (Zaber Technologies, Vancouver, Canada) was used to rotate the mice 360 degrees whilst breathing to obtain a 4-dimensional CT. The CT parameters used to obtain the images in this paper can be found in Table 1. Images were obtained at 30 frames per second (Hz) and image acquisition was triggered by the ventilator so that all images were acquired at the same points throughout the breathing cycle. This allowed all images to be binned to 15 time points over the respiratory cycle; in reference to the parameters used for Fig. 2 this provided 400 projections for each time point in the breath over 360 degrees for post-processing. The total CTXV image acquisition took ~3.5 minutes.

### Image reconstruction and analysis

A cone-beam reconstruction technique^26^ was used to produce CT reconstruction volumes for each time point in the breath, resulting in 15 complete volumes that each represent a different time point in the breath. A calibration scan of an acrylic cylinder with fiducial markers^27^ was imaged after each mouse to calculate the centre of rotation and projection angles used for the CT reconstruction.

Airways were segmented using an image filter based on Frangi *et al*.^28,29^. Local image gradients were matched to an ellipsoid, based on a Gaussian kernel scale, to differentiate tubular structures from plane-like structures. Tubular structures were computed over a range of 1–30 Gaussian kernel scales. This filter returns a probability value that any given pixel is part of a tubular structure, which, when segmented in Avizo (FEI VSG, France) using a flood-fill segmentation, returns a more accurate segmentation of the airways than segmenting the unprocessed CT volume alone. The airway segmentation was performed on the first of the 15 CT volumes (i.e. at the start of the breath before inspiration), because the volume of air throughout the distal gas exchange spaces is at a minimum at this time-point, creating maximal contrast to isolate the airways.

To extract functional respiratory data measurements (e.g. those represented in Fig. 3), the CT volumes were smoothed with a Gaussian filter to reduce noise, and the lung speckle visibility was enhanced using a bandpass filter. A 3-dimensional (3D) cross-correlation based technique was then used to measure the lung displacement between successive frames in the breath. The cross-correlation analysis was performed with interrogation regions of 32 × 32 × 32 voxels, with a 50% overlap between successive interrogation regions. The expansion field of the lung tissue was then calculated from the local gradients of the vectors. This information was displayed on a CT reconstruction of the lung tissue (see Fig. 3). The airflow through the airway tree was determined by associating the lung expansion data with the segmented airway tree through the Airway Tree Link (ATL) analysis method as described previously in Dubsky *et al*.^11^. As the air volume at the terminal airways can be calculated from the expansion data for the connected regional lung tissue, and the flow through the entire bronchial tree can be calculated by recursively summing the airflows in daughter segments at each bifurcation, with the assumption that the flow through a parent segment must equal the sum of the flow from the two supplying daughter segments and that airway compression effects are negligible due to the tidal breathing regime provided by the ventilator. This quantitative airflow measurement was then overlaid on the structural CT segmentation of the airways to visually present airflow through the bronchial tree (Fig. 2).

The expiratory time constant for the airway trees (Fig. 3a,b) is a standardised dynamic-change calculation that describes the time required for the lung volume to decrease by 63% (~1-(1/e), where e is the mathematical constant known as Euler’s number) of the complete expired volume and describes the system’s resistance and compliance. The Airway Tree Link analysis described above allowed the volume at each branch of the airway to be quantified, which in turn is then be used to calculate the time required for the local air volume in each branch to reduce by 63% of the tidal volume.

### Code availability

The 4DxV analysis code that supports the findings in this study is not publicly available due to patent restrictions. Code may however be available from the authors upon reasonable request and with permission of Monash University and 4Dx Limited.

## Data Availability

The data that support the findings of this study are available on reasonable request from the corresponding authors.
